# Migraine: A Review on Its History, Global Epidemiology, Risk Factors, and Comorbidities

**DOI:** 10.3389/fneur.2021.800605

**Published:** 2022-02-23

**Authors:** Parastoo Amiri, Somayeh Kazeminasab, Seyed Aria Nejadghaderi, Reza Mohammadinasab, Hojjat Pourfathi, Mostafa Araj-Khodaei, Mark J. M. Sullman, Ali-Asghar Kolahi, Saeid Safiri

**Affiliations:** ^1^Research Center for Integrative Medicine in Aging, Aging Research Institute, Tabriz University of Medical Sciences, Tabriz, Iran; ^2^Research Deputy, Faculty of Medicine, Tabriz University of Medical Sciences, Tabriz, Iran; ^3^School of Medicine, Shahid Beheshti University of Medical Sciences, Tehran, Iran; ^4^Systematic Review and Meta-Analysis Expert Group, Universal Scientific Education and Research Network, Tehran, Iran; ^5^Department of History of Medicine, School of Traditional Medicine, Tabriz University of Medical Sciences, Tabriz, Iran; ^6^Department of Anesthesiology and Pain Management, Faculty of Medicine, Tabriz University of Medical Sciences, Tabriz, Iran; ^7^Department of Persian Medicine, School of Traditional Medicine, Tabriz University of Medical Sciences, Tabriz, Iran; ^8^Department of Social Sciences, University of Nicosia, Nicosia, Cyprus; ^9^Department of Life and Health Sciences, University of Nicosia, Nicosia, Cyprus; ^10^Social Determinants of Health Research Center, Shahid Beheshti University of Medical Sciences, Tehran, Iran; ^11^Neurosciences Research Center, Aging Research Institute, Tabriz University of Medical Sciences, Tabriz, Iran; ^12^Social Determinants of Health Research Center, Department of Community Medicine, Faculty of Medicine, Tabriz University of Medical Sciences, Tabriz, Iran

**Keywords:** migraine, epidemiology, risk factors, comorbidity, narrative review

## Abstract

Migraine affects more than one billion individuals each year across the world, and is one of the most common neurologic disorders, with a high prevalence and morbidity, especially among young adults and females. Migraine is associated with a wide range of comorbidities, which range from stress and sleep disturbances to suicide. The complex and largely unclear mechanisms of migraine development have resulted in the proposal of various social and biological risk factors, such as hormonal imbalances, genetic and epigenetic influences, as well as cardiovascular, neurological, and autoimmune diseases. This review presents a comprehensive review of the most up-to-date literature on the epidemiology, and risk factors, as well as highlighting the gaps in our knowledge.

## Introduction

Migraine is defined as “an episodic headache associated with certain features, such as sensitivity to light, sound, or movement” or “a recurring syndrome of headache associated with other symptoms of neurologic dysfunction in varying admixtures” (1). Migraines can be placed into two categories, which are resistant migraines (i.e., “having failed at least 3 classes of migraine preventatives and suffer from at least 8 debilitating headache days per month for at least 3 consecutive months without improvement”) and refractory migraines (i.e., “having failed all of the available preventatives and suffer from at least 8 debilitating headache days per month for at least 6 consecutive months”) (2). Migraines can also be associated with different syndromes like somnambulism, cyclic vomiting, abdominal migraine, benign paroxysmal vertigo, benign paroxysmal torticollis and confusional migraine, which have different clinical presentations, duration and prevalence (3). Migraine is a cyclic disorder which has different phases, including a premonitory phase, transient neurological symptoms (i.e., migraine aura), intense headache attack and postdrome phase (4). Furthermore, migraine is a burdensome disease which has an impact on the individual's economic situation, family relationships, as well as work and school activities (5). Globally, migraine was the second largest contributor to the disability-adjusted life-years (DALYs) lost due to neurological disorders in 2016, accounting for 16.3% [95% uncertainty interval (UI): 11.7–20.8] of the attributable DALYs (6). The global age-standardized prevalence of migraine increased by 1.7% (0.7–2.8) from 1990 to 2019, and in 2019 there were 1.1 billion (0.98–1.3) prevalent cases and 525.5 (78.8–1,194) years lived with disability (YLDs) per 100,000 population (7). In the United States, the economic burden of migraine was significantly higher in patients with migraine, than among those without migraine ($11,010 vs. $4,436; *p* < 0.01) (8).

Based upon several proposed mechanisms for migraine (9), various risk factors have been identified, such as: advanced age, head trauma, lower socioeconomic status, caffeine or medication overuse, stress, sleep problems (e.g., snoring), obesity, pain syndrome, and pro-inflammatory or pro-thrombotic states (10). In addition to the above mentioned risk factors, several other risk factors have been suggested for chronic migraine, a subtype of migraine, including ineffective treatment of acute migraine and the overuse of medication (11). Demographic (e.g., sex and race) and lifestyle factors (e.g., caffeine misuse, body weight gain and sleep disorders) are also additional risk factors (10, 12).

Although previous review articles have proposed the risk factors for migraine progression or chronification (10, 11, 13), to our best of knowledge there have been no recent studies on migraine risk factors. Therefore, in this article we comprehensively review the most recent evidence on the risk factors for migraine, as well as describing the history of migraine.

## Methods

The PubMed database was searched for eligible studies up to August 21, 2021. We used “headache” OR “migraine” OR “headache disorder” OR “headache syndromes” key words combined with the Boolean operator AND for the following key words “biological factors” OR “age” OR “sex” OR “gender” OR “hormone” OR “genetic” OR “epigenetic” OR “comorbidity” OR “alcohol” OR “education” OR “fatigue” OR “patient education” OR “perfectionism” OR “psychiatric disorders” OR “psychological disorders” OR “psychological factors” OR “sleep disorders” OR “sleep problems” OR “smoking” OR “social factors” OR “stress” OR “mood disorders” OR “mental disorders” OR “mindfulness” OR “obsessive-compulsive disorder” OR “panic disorder” OR “patient education” OR “personality disorders” OR “personality traits” OR “phobia disorder” OR “post-traumatic stress disorder” OR “neuroticism.” Our query for assessing the history of migraine was (“migraine” OR “headache”) and (“history” OR “history of medicine”).

Any type of primary or secondary studies that evaluated the relationship between different disorders or agents with migraine in humans were included in this study, but there were no filters on publication date, article type, or sex. Studies were excluded if they were not in English, were *in-vitro* or were conducted on animal, or evaluated the risk factors for other types of headaches (i.e., other than migraine). Firstly, two independent authors screened the title and/or abstract of the identified studies and then full-texts were obtained for all articles that made it through the first screening process and these were also screed by the two authors. At both steps, any discrepancies between the reviewers were resolved by discussion or through consultation with a third author.

## A Brief History of Migraine

Migraine, and the associated pain, has long plagued humans (14). Many people have suffered from migraines, including Julius Caesar, St. Paul, Thomas Jefferson, and Charles Darwin (15). Three thousand years BC, an anonymous Sumerian poet described a state of illness in which the headache was so severe that the patient wished to be relieved of the pain by death (14, 16). In Mesopotamia, it was believed that the evil spirit of Ti'e was attacking its victims, leading to terrible headache symptoms (14, 15). Skulls have been found in archeological excavations, which indicate that doctors recommended removing parts of the patient's skull to relieve severe headaches (14, 15).

In ancient Egyptian medicine (From 3,000 BC), migraines and nerve pain, as well as sudden and severe pain, were clearly described (17). Hippocrates (460 – 370 BC), the father of medicine, was the first to scientifically document his clinical observations about migraine, freeing the disease from superstition (18, 19). Hippocrates described a bright light in the right eye, followed by the onset of severe pain in the head, which eventually extended to the entire area (18, 19).

Aretaeus of Cappadocia (30–90 A.D.) provided one of the first formal classifications for headaches (20, 21). He divided headaches into three main types: (a) cephalalgia, a mild and short-term headache, (b) cephalea, a type of headache that is chronic and more severe, and (c) heterocrania, a paroxysmal headache on one side of the head, often accompanied by nausea, vomiting, photophobia, symptoms of the eyes, sweating and changes in the perception of fragrances (20, 21).

Galen (129–216 AD) wrote about a severe pain that affected almost half of the head (22, 23). Galen, like Hippocrates, believed that migraines were caused by vapors rising from the stomach into the head (22, 23). Razi (also known by his Latinized name Rhazes, 854–925 CE), a famous Iranian physician (24), devoted an entire chapter to headaches in his book, in which he wrote about the symptoms and treatment of migraine. His work described in detail one of the different types of headaches, now known in modern medicine as migraine clusters, and he also suggested that severe and sudden headaches may be a preliminary sign of a stroke (25).

Ibn Sina, also known in the West as Avicenna (980–1,037), another famous Iranian physician (26, 27), wrote a chapter on headaches in his book “Canon of medicine,” which described different types of headaches, including one type which covered the entire head. This type of headache was severe, intensified by activity and was also characterized by photophobia and noise phobia (28–30).

In the 17th century, Thomas Willis (1,621–1,675) published his hypothesis that “megrim” was due to the dilatation of blood vessels within the head (the first enunciation of a vascular theory) (31). In 1,873, Edward Liveing proposed that migraine was due to “nerve storms evolved out of the optic thalamus” (32). In 1,898, Moebius stated that “parenchyma is the master, circulation the servant,” and that both brain and blood vessel dysfunctions were necessary to produce a migraine attack (32, 33).

## Global Epidemiology and Trends

Migraine is one of the most common neurological diseases worldwide, with the global prevalence of migraine being estimated to be 1.1 billion [95% uncertainty interval (UI): 0.98–1.3] cases in 2019 (7). In that same year, the national age-standardized point prevalence of migraine ranged from 8,277 to 22,400.6 cases per 100,000 (7). The highest age-standardized point prevalence rates were found in Belgium [22,400 (95% UI: 19,305.2–26,215.8)] and Italy [20,337.7 (95% UI: 17,724.7–23,405.8)], while the lowest rates were found in Ethiopia [8,277 (95% UI: 7,226.2–9,527.5)] and Djibouti [8,915.3 (95% UI: 7,629.9–10,476.6)] (7).

In 2019, the national age-standardized incidence rates of migraine ranged from 692.6 to 1,528.4 cases per 100,000, with Italy [1,528.4 (95% UI: 1,345.4–1,709.3)] and Norway [1,515.7 (95% UI: 1,333.8–1,693.4)] having the highest rates. In contrast, the lowest rates were observed for Ethiopia [692.6 (95% UI: 605.2–776.7)] and Djibouti [737.2 (95% UI: 623.5–848.9)] (7).

The global prevalence of migraine has increased substantially over the last three decades (7). According to the Global Burden of Disease (GBD) 2019 study, the estimated global prevalence of migraine increased from 721.9 million (95% UI: 624.9–833.4) in 1990 to 1.1 billion (95% UI: 0.98–1.3) in 2019 (7). The percentage change in the global age-standardized prevalence rate and years lived with disability (YLDs), from 1990 to 2019, were 1.7 (95% UI: 0.7–2.8) and 1.5 (95% UI: −4.4 to 3.3), respectively (7). During this period, the highest increases in the age-standardized prevalence per 100,000 population were in East Asia (7.9% (95% UI: 4.3–12%) and Andean Latin America [6.7% (95% UI: 2.1–11.9%)], while the largest decreases were in High-income North America [−2.2% (95% UI: −5.3 to 1.1%)] and Southeast Asia [−2.2% (95% UI: −3 to −1.4%)] (7). Furthermore, the age-standardized YLD rate of migraine also increased from 517.6 (95% UI: 82.0–1169.1) in 1990 to 525.5 (95% UI: 78.8–1194.0) in 2019 (7).

The prevalence of migraine was higher in females, than in males, across all age groups. In 2019, the global age-standardized prevalence rate in females and males were 17,902.5 (95% UI: 15,588.3, 20,531.7) and 10,337.6 (95% UI: 8,948.0, 12,013.0) per 100,000 populations, respectively (7).

The highest incidence rate and number of incident cases of migraine were in the age group 10–14 years, in both females and males (7). In 2019, while the number of YLDs started increasing from birth, they peaked in the 30–34 age group and then gradually declined for both sexes (7).

Socioeconomic status did not seem to be associated with the burden of migraine, since no clear association was reported between the socio-demographic index (SDI) and the migraine YLD rate (7).

## Biological Risk Factors and Comorbidities

Large-scale epidemiological studies have identified several risk factors for migraine attacks. For instance, a web-based survey of 15,133 patients with migraine and 77,453 controls showed that insomnia, depression, anxiety, gastric ulcers and/or gastrointestinal bleeding, angina and epilepsy were significantly higher among migraineurs than among the control group (*p* < 0.001) (34). Also, the intensity and frequency of pain are both related to biological and psychological disorders, as well as due to inflammation (34). In accordance with the complex nature of migraines, differentiating between migraine risk factors, triggers, and outcomes is difficult (13). Informing patients about the causes and exacerbating factors can reduce the frequency and severity of attacks (13). Furthermore, some interventions, such as aerobic exercise, can reduce migraine attack duration, pain intensity and decrease the number of migraine days/month (35). However, based upon current knowledge, migraine shows a wide range of risk factors, triggers, and comorbidities (10, 13). Table 1 shows the different risk factors associated with migraine development and progression.

**Table 1:** Risk factors for migraine.

Biological factors	Hormonal imbalances	Estrogen dysregulation	
			Cortisol dysregulation
		Demographic factors	Advancing age
			Female sex
		Metabolic factors	Obesity
			Dyslipidemia
			Diabetes
			Hypertension
		Genetic and epigenetic factors	*MTDH* gene
			*MEF2D* gene
			*PRDM16* gene
Psychological factors	Psychological factors	Anxiety	
			Phobia
			Panic
			Stress
Miscellaneous^*^	Miscellaneous^*^	Lower level of education	
			Lower socioeconomic status

* *These conditions need to be furtherly assessed by future studies*.

### Hormonal Imbalances

Migraine attacks are often associated with fluctuating hormone levels (36, 37). Hormones control chemicals in the brain that affect the sensation of pain, so any hormonal imbalances can influence the pain-processing networks in the brain (36, 37). Hormones have a potential connection to the pathophysiology of migraines (36). Of the hormones investigated, most of the attention has been directed to researching the level and fluctuations of sex hormones (37). In this section, we review the role of hormones in migraines.

#### Estrogen

Females suffer from migraines at about twice the rate of men (38). In addition to the prevalence rate, sex-based differences in migraine attacks have been validated by studies using structural and functional magnetic resonance imaging (MRI) of the brain (39). Alterations in the posterior insula and the thickness of the precuneus cortices have been identified in female migraineurs, in comparison to males and healthy controls from both sexes, using structural MRI scans (39). Functional MRI studies have also found evidence of gender differences in pain sensitivity and pain processing pathways (40). Although, both biological and psychosocial factors contribute to the sex differences in migraine, it seems that sex hormones are the most important (41). Migraine occurrence can be affected by menstruation, pregnancy, and menopause, in addition to the use of hormonal contraceptives and hormone replacement therapies (39). In this regard, a recent systematic review of 12 studies showed that high-estrogen levels, high estrogen fluctuations, and hormonal replacement therapy were associated with the worst migraine outcomes (42).

Menstrual migraine is a specific condition when the timing of the attacks are associated with drops in estrogen levels a few days before menstruation (40, 43). There are close interrelationships between estrogen and several neurotransmitters, including serotonin, norepinephrine, dopamine, catecholamines, and endorphins (40, 43). The results from positron emission tomography (PET) scans show that fluctuations in estrogen have a powerful influence on the balance of the previously mentioned neurotransmitters, which all play a crucial role in the communication between nerve cells (40, 44). Therefore, estrogen administration to premenstrual and menopausal women can decrease the occurrence of migraine headaches (40, 45, 46). Nevertheless, there have been controversial findings regarding the administration of low doses of estrogen to female migraineurs and this treatment also raises the risk of stroke, cardiac disease, and vascular mortality (43, 44).

#### Cortisol

Cortisol is a steroid hormone produced by the adrenal gland in response to stress (47). Elevated cortisol levels create physiological changes and effects a wide range of processes, such as body metabolism, blood pressure, and the immune system (47). One likely hypothesis is that the increased levels of cortisol can cause migraines (47). Another hypothesis is that the increased levels of the stress hormone cortisol, during the early morning, may be responsible for the circadian variation in migraine attacks (48). Surprisingly, these findings were not confirmed in the latest systematic review of the research (47, 48), which found no significant association between cortisol levels and the pathogenesis of migraine (47) or between circadian variations and the onset of migraine attacks (48).

#### Thyroid Hormones

A bidirectional relationship between migraine and hypothyroididsm has been suggested (49). Migraines may be as a result of high thyroid stimulating hormone (TSH) levels which can lead to pituitary growth and the compression of intrasellar structures (50). Furthermore, a systematic review of case-control studies showed that the weighted overall standardized mean difference for serum levels of TSH, T3, and T4 in migraineurs, compared with non-migraineurs, were 0.80 (95% CI: 0.04, 1.56), −0.27 (95% CI: −0.66, 0.12), and 0.09 (95% CI: −0.08, 0.26), respectively (51). Therefore, migraine and hypothyroidism can be considered to be comorbid (49).

### Metabolic Factors

Metabolic syndrome (MetS) is a cluster of metabolic disorders that includes abdominal obesity, diabetes, dyslipidemia, and hypertension (52). Increasing urbanization, hypernutrition, sequential abdominal fat accumulation, and sedentary life styles are the greatest known risk factors for MetS (52). However, a number of studies in different populations have made conflicting findings about the associations migraine has with obesity, diabetes, hypertension, and hypothyroidism (49, 53–59).

While obesity is considered a risk factor for episodic and chronic migraines, this association is most commonly observed in women under the age of 55 years old (53). Therefore, it seems that the association between obesity and migraine is dependent upon gender and age (53). Moreover, several studies have investigated the relationship that migraines have with adipocytokines (i.e., cell signaling proteins that participate in glucose, lipid, and energy homeostasis), but their conflicting findings preclude any solid conclusions (54).

The results of a systematic literature review have shown the protective effect of type 1 diabetes on migraines, but there was no significant relationship between migraine and type 2 diabetes (55). The association between migraine and the risk of developing type 2 diabetes was evaluated in a prospective cohort study, which found that the development of type 2 diabetes was reduced in women with active migraines (56). The bilateral association between diabetes and migraine has also been shown in a systematic review and meta-analysis (57).

Several studies have found hypertension to be associated with migraine, especially in older adults (58–62). Uncontrolled hypertension has been reported as a risk factor for migraine, with and without aura, and hypertension may also induce migraine chronification (34, 55, 58–61). Hypertension also increases the risk of cerebrovascular and cardiovascular complications among migraineurs (62). However, it seems that the association between migraine and hypertension may be due to the fact that both are closely related to several other risk factors, such as environmental factors and genetic vulnerabilities (34, 62).

Dyslipidemia is one of the factors involved in the development and intensification of migraine attacks and also contributes to the increased risk of cardiovascular disease (63). An analysis of the lipidomic profiling of migraine patients' plasma revealed a significant association with elevated levels of high density lipoproteins (HDL) (63). Furthermore, research has found a male-specific association between migraine status and omega-3 fatty acids (63). Moreover, a recent systematic review found elevated total serum cholesterol to be a risk factor for migraines (34).

Currently available evidence appears to confirm that a complex relationship exist between migraine and MetS (64). However, there is a need for more robust studies, using larger groups from the general population, to determine the biological mechanisms underlying the metabolic alterations in migraine patients.

### Genetic and Epigenetic Factors

Epidemiological research has found that genetic factors play a substantial role in the development of both migraine with aura and migraine without aura (65, 66). The concordance rate in monozygotic twins is higher than in dizygotic twins. The evidence also implies that migraine is a heritable trait and data indicates 42% heritability (95% CI: 36–47%) (67). This finding was also supported by the analysis of polygenic risk scores (PRS), which confirmed the familial aggregation of migraine (68, 69). The severity of migraines, and early age of onset, are associated with high PRS in familial cases (68, 69).

Familial hemiplegic migraine (FHM) is a monogenic autosomal dominant subtype of migraine with aura, which is caused by mutations in three genes, which are *CACNA1A, ATP1A2*, and *SCN1A* (70–72). All three are ion transport-related genes, which control neuronal activity via the modulation of glutamate availability at the synaptic terminals (73).

The polygenic nature of complex traits and disorders explains the small, but additive effect of many genetic loci in the pathophysiology of migraine (74). Over the past decade, genome-wide association studies (GWAS) have used high-throughput genotyping technologies to identify the associated loci and variants with migraine risk. The first GWAS on migraine was conducted in 2010, which identified rs1835740 as the first genetic risk factor for migraine (74). In the GWAS conducted to date, increasing the sample size and international collaborations has led to remarkable progress in the identification of the novel genetic variants associated with migraine (75–78). The results of 22 GWAS from the International Headache Genetics Consortium (IHGC) were combined to identify new susceptibility loci for migraine (79). Thirty-eight distinct genomic loci were identified, 28 of which had not been previously reported (79). Expression analysis of the identified genes demonstrated the two most strongly enriched tissues, which included the aorta and tibial artery in the cardiovascular system, and the smooth muscles of the esophagus and the esophageal mucosa in the digestive system (79).

The tissue-specific pattern of epigenetic modifications, and the restricted access to several tissue types of the body, have limited the study of the epigenetic process (80, 81). However, there is some evidence that the variants associated with phenotypes expressed in inaccessible tissues, such as the brain, can also be detected in the blood (80, 81). Therefore, in recent decades the pivotal role of epigenetics has been investigated in the development of complex diseases in the human population (80). DNA methylation is the principal epigenetic tag found in the DNA sequence (80). Epigenetic factors can affect migraine development, chronification, and even the response to treatment (80). Mutations in the epigenetic effector protein methyl CpG binding protein 2 (MeCP2) is associated with Rett syndrome (82) and mutation in ATRX is associated with alpha thalassemia (83). Both of these can contribute to epilepsy, which is one comorbidity of migraine (80). Moreover, epigenetics can explain the migraine chronification by brain epigenome alteration, due to increased neuronal activity in migraineurs (80). According to animal models for depression, HDAC activity should be decreased by antidepressant drugs to achieve the chronic stress effects on epigenome (84). Valproate, as a HDAC inhibitor, can facilitate the process (80). GWAS have identified the association between migraine and genes that appear to be involved in the epigenetic processes, including *MTDH, MEF2D*, and *PRDM16* (80). Epigenome-wide association studies (EWAS) have revealed migraine-associated regions that modify disease susceptibility by altering the expression levels of their target genes (81).

### Smoking, Alcohol, and Substance Abuse Disorder

Previous research has identified an association between migraine and the increased risk of substance abuse and addiction (85). Compared to major depression and anxiety disorders, the comorbidity between substance abuse and migraine is weaker and the findings are sometimes contradictory (86). Since substance abuse also has high comorbidity with BD (bipolar disorder), it is possible that the apparent relationship between migraine and substance abuse could potentially be accounted for by the comorbidity between migraine and BD (86).

Alcohol is one of the top 10 migraine triggers, and has been shown to increase the risk of migraine development by up to 51% (87). However, there is also evidence which has found no link between alcohol and migraines (85) and there remains some uncertainty about the mechanisms by which alcohol triggers migraine attacks (88).

### Age and Sex

Age, female sex, and low educational status are the most important demographic risk factors for migraine chronification, while higher education can act as a protective factor against migraines (11). Migraines can develop at any age, but the prevalence rate appears to be highest before the age of 45 (89). The prevalence rate rises throughout early adult life, peaking during middle age and then falls gradually (89).

Multiple epidemiological studies have found significant sex differences in the prevalence of migraines, with three times as many women affected as men (7, 90). The findings from structural and functional brain MRIs have confirmed sex-related differences in migraine attacks (41). The higher prevalence of migraines in women could be related to several biological and psychological differences between men and women, such as sex hormones, genetic factors, exposure to environmental stressors, as well as the level and response to stress and pain (90, 91). However, the increased prevalence of migraines with the onset of puberty and menarche, during menstruation, pregnancy, and menopauses suggests that fluctuations in sex hormones, especially estrogen, play a critical role in the higher prevalence of migraines in women (90, 92).

### Sleep Disorders

There is a strong and significant relationship between sleep and migraines, in which sleep disturbances are associated with migraine type and severity (34, 91). Migraine is associated with different types of sleep problems, like insomnia, sleep-related breathing disorders (e.g., obstructive sleep apnea), sleep-related movement disorders (e.g., restless-leg syndrome), central disorders of hypersomnolence (e.g., narcolepsy), circadian rhythm-related sleep-wake disorders and parasomnia (93). Furthermore, sleep complaints occur with greater frequency among chronic, rather than episodic, migraineurs (94). Sleep problems are also one of the most common factors precipitating migraines (8.3–64%) (87, 95). Disturbances or changes in sleep rhythms are among the most frequently cited migraine triggers and can lead to a full migraine attack (11, 87, 96). The results of a meta-analysis showed that people with migraines, compared to non-migraine sufferers, were more than three times as likely to experience insomnia (34). Nonetheless, the relationship between sleep and headache is a complex and bidirectional relationship. Sleep disturbances may result in headaches and headaches may result in sleep disturbance, or sleep disturbances and headaches may occur simultaneously, because both are related or because they are symptoms of another disorder (97).

### Fatigue

Fatigue and chronic fatigue syndrome (CFS) are common in patients with chronic migraines (98). Physical fatigue is often associated with the precipitation of migraine attacks (87). Although the relationship between fatigue, stress, and migraines is complex, migraineurs often report feeling a significant decrease in what they perceive to be a normal degree of physical strength and/or energy (87).

### Eating Disorders

Eating disorders have also been found to be associated with migraines (99). Eating disorders may be characterized by specific behaviors, such as avoidance fasting and skipping meals, that may trigger a migraine (88). Eating disorders, including anorexia nervosa (AN) and bulimia nervosa (BN) are severe psychiatric and somatic conditions that occur mainly among young women (88). However, whether eating disorders are linked to migraine, remains somewhat controversial (88). Although the causes of eating disorders are largely unknown, there is evidence to suggest that biological and psychological factors play an important role in the pathogenesis, along with monoamine, indole, and same hypothalamic centered hormonal disorders (89, 100). Migraine is characterized by similar metabolic and psychological anomalies, suggesting that a possible association between the two pathological conditions exists (100). A study by Gebhardt et al. showed that women who had been diagnosed with an eating disorder during their lifetime were twice as likely to also report having a history of migraines, as women from the same cohort without an eating disorder (88). Mustelin et al. hypothesized that this relationship was via the comorbidity that both migraine and eating disorders had with major depression disorder (MDD) (101). Thus, eating disorders may enhance the likelihood of developing a migraine, in a specific subgroups of subjects, possibly through the influence of other factors, such as anxiety or depression (88). In contrast, a clinic-based investigation found no evidence of an increased prevalence of migraine among patients with eating disorders (102). Nevertheless, specific manifestations of eating disorders, such as dieting, fasting, or skipping meals, are often reported as migraine triggers (87, 88).

According to a recent review, the Ketogenic diet and modified Atkins diet are both helpful for migraine prevention and control, playing important roles in neuroprotection, improving energy metabolism and mitochondrial function, decreasing calcitonin gene-related peptide levels and the suppression of neuroinflammation (103). A balance between the intake of essential fatty acids, omega-6 and omega-3, as well as applying dietary strategies for weight reduction can play roles in migraine prophylaxis (103).

### Cardiovascular Diseases

Currently, migraine is considered to be a risk factor for cardiovascular disease (104). This connection is stronger in patients who experience aura than in those without aura (104). Generally, migraines and cardiovascular diseases involve changes in blood flow, meaning that the comorbidity between cardiovascular diseases and migraines may be due to insufficient blood flow to the brain and heart as a result of a genetic predisposition, hormonal imbalance, endothelial dysfunction, coagulation abnormalities, or arterial dissection (104, 105). Polygenic risk analysis of genome-wide association studies, which focused on candidate genes, revealed the predisposing role of genetics in the development of both cardiovascular diseases and migraine (106). Gene expression enrichment analyses have also confirmed there to be a shared biological basis between migraine and coronary artery disease (34, 104, 106–108).

Evidence suggests that patients who have migraine with aura have a higher risk of ischemic stroke, hemorrhagic stroke, and subclinical ischemic lesions (109). All of these types of strokes are classified as migraine-related strokes (109). Studies also indicate that age, being female, smoking, and oral contraceptive use are potential risk factors for ischemic stroke in migraine sufferers (109).

Numerous studies have also shown migraine to be an important risk factor for ischemic heart disease (IHD), as well as other adverse cardiac events, such as myocardial infarction, angina, and arrhythmia (110, 111). Furthermore, research has also found an increased risk of patent foramen ovale, atrial septal defect, and mitral valve prolapse in migraine patients with aura (110, 111).

### Movement Disorders

Clinical and experimental research on the interactions between migraine and the extrapyramidal system, which is a part of the motor system network causing involuntary actions, confirmed a higher prevalence of migraines in patients with basal ganglia disorders (112). The basal ganglia has an important role in the pathophysiology of some chronic pain disorders, including migraines (112). The evidence and possible pathological mechanisms justify the comorbidity of migraine with some basal ganglia disorders, such as restless legs syndrome, Tourette's syndrome, Parkinson's disease, essential tremors, dystonia, and Sydenham's chorea (112). The high prevalence of migraine among individuals with movement disorders highlights the role of common pathogenic pathways, such as the serotonin system dysregulation in Tourette's syndrome and dopaminergic dysfunction and dysfunctional brain iron metabolism in both restless leg syndrome and Parkinson's disease (113–116).

### Neurological Disorders

The available evidence confirms the co-occurrence of several neurological disorders with migraine (89). This fact highlights the role that the same biological pathways have in the pathogenesis of these disorders. However, similarities in the symptoms of neurological disorders and migraines can also lead to misdiagnosis. As a result, it seems likely that raising awareness about these comorbidities among health care providers can help to enable better management of these patients. There is also evidence that migraines have a relationship with both epilepsy and multiple sclerosis (117, 118).

#### Epilepsy

Migraine and epilepsy are distinct neurological disorders, with one of the hallmark of both being their paroxysmal nature (119). Moreover, there are several overlaps in the triggering factors, clinical symptoms, and pathogenic mechanisms (119). Some systemic symptoms, such as nausea, vomiting, visual and other sensory disturbances, such as olfactory dysfunction are common in both migraines and epilepsy (117, 119, 120). Neurogenic and neurovascular findings have found that migraine attacks, like epileptic seizures, can originate from electrical disturbances in the brain (117, 121). Evidence also shows that neuronal over activity in migraine leads to cortical spreading, depression and aura, and that excess neuronal activity leads to the recruitment of larger populations of neurons firing in a rhythmic manner that eventually leads to epileptic seizures (117, 121). The prominent role of electrical transmembrane gradients in the pathogenesis of both migraine and epilepsy can be explained via a genetic overlap of different mutations in the ion channels and neurotransmitter receptor genes (117, 120–122). The co-occurrence of FHM and epileptic seizures or the occurrence of seizures during migraine attacks (i.e., migralepsy), confirms the shared genetic basis of the two disorders (121, 123). Just as similar biological mechanisms are involved in the development of attacks, it would not be surprising if similar medications were effective in preventing or treating the attacks for these two disorders (122).

#### Multiple Sclerosis

MS is the most common chronic inflammatory disease of the central nervous system (124). Headache, especially the migraine subtype, is frequently an early symptom of MS (124). A recent global survey has confirmed the comorbidity between migraine and MS (118). The shared mechanisms underlying their comorbidity are not completely understood, although genetic factors, neuroimmunological and inflammatory mechanisms can explain their comorbidity (124, 125).

### Autoimmune Diseases

Recent experimental and epidemiological evidence, such as similar age of onset, remission, gender-specific prevalence, and imbalanced T-cell immune status, appear to suggest that migraine is an autoimmune disease (126, 127). Furthermore, there is comorbidity between migraine and autoimmune diseases, such as rheumatoid arthritis and psoriasis (128, 129).

#### Rheumatoid Arthritis

RA is a chronic autoimmune disease, marked by symptoms of inflammation and pain in the joints (128). Therefore, chronic pain, such as migraine headaches, are hallmark symptoms of RA (128). The results of population-based cohort studies have confirmed the comorbidity of RA in those suffering from migraine (34, 128). A recent biocomputational genetic association analysis of migraine and RA demonstrated that there is a phylogenetic relationship between the genes responsible for migraine and RA, such as *SLC24A3* and *HLA-B, MPPED2* and *STAT4*, and *ATP1A2* and *IL6R*, respectively (130). Therefore, this common ancestry and genetic similarity can explain the comorbidity of migraine and RA (130).

#### Psoriasis

Psoriasis is a long-lasting autoimmune disease that causes raised, red, and scaly patches to appear on the skin (129). The research evidence has shown that patients with psoriasis have a higher chance of developing migraines (34, 129). Although the exact mechanism by which psoriasis increases the risk of migraine remains unclear, it seems that neurogenic inflammation plays an important role in the link (131, 132). Proinflammatory cytokines, such as tumor necrosis factor (TNF)-α, interleukin (IL)-12, and IL-23, are produced predominantly through the activation of the plasmacytoid dendritic cells (133, 134). These proinflammatory cytokines can trigger the development of psoriasis through systemic inflammation (133, 134). The increased TNF-α expression induces endothelial dysfunction (133, 134). Likewise, neurogenic inflammation is generated by the release of neuropeptides, which can lead to inflammation and the release of proinflammatory cytokines, such as TNF-α (133, 134). It has been proposed that neurogenic inflammation plays a pivotal role in the generation of pain in migraine headaches (133, 134).

### Gastrointestinal Disorders

The gut-brain axis, which is referred to as the interaction between the gastrointestinal system and the central nervous system, can affect migraine development and progression (135). There are a number of factors that play roles in this process, like inflammatory mediators (e.g., IL-6, IL-8 and TNF-α), the profile of gut microbiota, stress hormones, neuropeptide and serotonin pathways, in addition to nutritional constituents (135). Infection with *Helicobacter pylori* has also been found to be associated with migraine, as a meta-analysis of case-control studies showed that migraineurs had a significant higher prevalence of *H. pylori* than the controls (44.97 vs. 33.26%; *p* = 0.001) (136). Moreover, irritable bowel syndrome has a statistically significant relationship with migraine (137, 138). Furthermore, a bidirectional association has been found between migraine and celiac disease (139, 140).

## Psychological Risk Factors and Comorbidities

The relationship between migraine and certain psychological factors, such as the tendency toward perfectionism, neuroticism, repressed aggression, and a melancholic mood have been reported for over a century (141). On a theoretical level, triggers raise intriguing questions about the nature of migraines (142). Clinically, triggers seem to be additive, with a migraine resulting when a particular threshold is breached (142). In the laboratory, isolated triggers tend to have less effect than exposure to several triggers in succession (142). This implies that triggers are converging using a common pathway, but the mechanism by which, for instance, psychological, social, occupational, genetic, and nutritional factors interact needs further investigation (142).

### Stress

Increasing stress levels contribute to a higher prevalence of migraine (143). In fact, stress may be the most important factor in triggering migraine attacks 97)). However, it should be noted that the word stress means different things to different people and can also be described using various synonymous words. About 70 years ago, Dr. John Graham hypothesized that “migraine is an inherited disorder occurring in people who have both an undue tendency to seek stress and also have a deficiency in their physiological adaptation to stress.” This hypothesis has been shown to be correct by a systematic review which was conducted in 2014 (87).

In a study on the prevalence of various migraine triggers among 494 patients, stress was found to be the most common migraine trigger (62%) (144). In another study of 71,877 migraineurs, 58% reported stress as the most important migraine trigger (87). Migraineurs may simply respond to stress with a headache and intense stress is common as an initiating factor for the first migraine attack (144). An article by Lantéri-Minet et al. demonstrated that the comorbidity of stress with migraines has both a statistically significant and clinically important detrimental effect on patients' quality of life (145). According to a review, young headache patients are more often afflicted by stress in their daily lives (146).

### Anxiety Disorders

Anxiety disorders include generalized anxiety disorder, phobia, panic disorder, and social anxiety (147). Several studies have shown that individuals with migraine are at an increased risk for affective and anxiety disorders (34, 141, 148–151). Among these psychiatric disorders, anxiety is a common comorbidity in migraine patients (91, 152–154). In a meta-analysis conducted on 15,133 migraine sufferers and 77,453 healthy controls, migraine sufferers were three times more likely to experience anxiety than non-migraine sufferers [odds ratio (OR) = 3.18; 95% confidence interval (CI): 3.0, 3.3] (34). The odds of experiencing anxiety were highest in the younger age groups, while being male, being married, being employed, and having a higher household income were associated with a lower risk of anxiety (34).

### General Anxiety Disorder

GAD is a common anxiety disorder that includes persistent and chronic worrying, nervousness, and tension (155). It is characterized by the presence of pervasive anxiety and repetitive worries about specific events (155). The prevalence of GAD is higher in subjects with migraine than in those without (88, 156). In fact, migraineurs have a 4–5 times higher risk of GAD than individuals without migraine (157). The relationship between migraine and GAD appears to be a reciprocal relationship. Not only are migraine headaches reported more often in patients with GAD, but the presence of GAD often precedes a migraine diagnosis (151).

Previous research has also found that GAD patients differed from the control group in the frequency of migraine headaches, but not in the frequency of tension-type headaches (TTH) (151). This finding is in agreement with the results of previous research on affective disorders (148, 149), which have found a bidirectional relationship between affective disorders and migraine (143), while that relationship between affective disorders and TTH does not exist, or is at best unclear (143). A cross-sectional survey of adults (18–65 years) in 10 European Union (EU) countries found that depression, especially anxiety, had significant comorbidity with migraine (158).

### Panic Disorder

PD is an anxiety disorder in which the patient suffers from panic attacks, which are sudden feelings of terror when there is no real danger (155). Individuals with migraines are between 1.2 and 10 times more likely to suffer from PD than those without migraine (88, 147, 159), with the highest ORs being for migraine with aura (147). However, these findings contrast slightly with research which has shown phobic and panic disorders to be more common among patients suffering from migraines without aura (160). All cross-sectional studies on the prevalence of psychiatric disorder in migraine patients, compared with individuals' without migraine, have found migraine sufferers have an increased risk of anxiety disorders, particularly PD and phobias (141).

According to the literature, PD normally appears earlier in patients with migraine, compared to people without migraines (88). However, like many other anxiety disorders, the relationship between PD and migraine is likely to be bidirectional (141), with the relationship being primarily from migraine to PD, although a weaker significant relationship has also been observed in the opposite direction (88). Migraine increased the risk for the first onset of PD, and PD increased the risk for the first onset of migraine (147). Also, during the follow-up period, the risk of panic disorder and phobia onset were greater for patients with migraine, than for those without (141). The data suggests that the temporal relationship is in the migraine-to-panic disorder direction, rather than the reverse, and also the comorbidity with panic disorder is not specific to migraine, but also includes other headache types (161).

### Phobia

Phobia is an anxiety disorder that has been studied in association with migraine (155). Some cross-sectional investigations have confirmed an increased risk of phobias in patients with migraines, compared with non-migraine sufferers (86, 162). Specific phobias are more prevalent in people with migraine, than they are in the general population (91). A review study demonstrated that among the various types of anxiety disorders, phobias and panic disorder had the highest comorbidity with migraines (86). Furthermore, it appears that while migraine with aura is more commonly comorbid with anxiety disorders, depression, and hypomania, PD and phobia are more common in migraine patients without aura (160). Nevertheless, research has not found a relationship between agoraphobia and migraine, which may be due to the rarity of this disorder (161). Furthermore, the same study also reported that social phobia, one type of anxiety disorder, exhibits the greatest association with migraine (161).

### Obsessive-Compulsive Disorder

Migraine sufferers are five times more likely to develop OCD, in comparison to individuals without migraine (157), although the results on the association between OCD and migraine are inconsistent. Some investigations have confirmed the association between OCD and migraine (141, 163), but subsequent investigations did not replicate these findings (141).

### Post-traumatic Stress Disorder

An association between PTSD and migraine has also been recently recognized (164). The prevalence of PTSD has been found to be higher in migraineurs, whether episodic or chronic, than among the general population (91, 165). The lifetime prevalence of PTSD in episodic migraine patients is more than four times that of the general population (164).

The presence of PTSD is independently associated with greater headache-related disability in migraineurs (165). The importance of the link between stress and migraines is that migraineurs with PTSD, compared to those without PTSD, were found to have more difficulties with developing and maintaining a social life, have a greater disability and lost more workdays due to physical health, mental health or substance abuse (8 vs. 2.6 days) (85). Central sensitization and neurotransmitters, like serotonin, play an important role in the pathophysiology of PTSD (88).

### Personality Traits

Several studies have found migraines to be associated with certain personality traits, such as neuroticism, hypochondriasis, depression, hysteria, and schizophrenia, but it is not yet clear whether these traits predispose a person to migraines simply as a result of the stress and inconveniences caused by these personality characteristics (166). Headache is a common complaint among individuals with personality disorders who seek emergency treatment, and these disorders make treatment more difficult (160). However, there are a number of personality traits that are more prognostically important (11, 166). For example, the wide personality trait of neuroticism has been found to be comorbid with both depression and migraine (34, 88).

In addition to personality disorders, subclinical personality traits have been found to be associated with migraines (167). Early studies reported that migraineurs' personalities appeared to be characterized by orderliness, perfectionism, inflexibility and a tendency to react excessively to problems, which in turn could lead to migraine attacks (167). Nevertheless, there is also evidence to suggest that there are no dominant personality profiles among migraine patients, although personality disorders are likely to complicate headache treatment (88, 168, 169). In studying the relationship between personality and migraine, it is also important to consider individual personality characteristics (e.g., tendency to perfectionism) (141). In general, the findings regarding the relationship between personality traits and migraine are contradictory and complex. However, it has been suggested that clinicians take personality traits into consideration, as they may influence the success of any treatment (88).

### Bipolar Disorder

BD is a chronic disease characterized by repeated manic (or hypomanic) and depressive episodes alternating over a long period of time (155). BD is one of the disorders that has a comorbidity with migraine (11, 34, 141, 170). Patients with BD show an increased prevalence of migraine, which has been reported in up to 55.3% of patients (88) and most often migraine precedes the onset of BD (171). There is research that suggests the prevalence of migraine is higher in subjects with both manic and depressive episodes, than it is among those only with depressive episodes (88). Compared to major depression, the relationship between migraine and bipolar spectrum disorders is about three times higher, with a stronger relationship for migraine with aura than for migraine without aura (147). The comorbidity of migraine and depression made individuals more susceptible to BD, so migraine in depressed patients might be a bipolar spectrum trait (147, 172). The mechanism of comorbidity between BD and migraine may be explained by heritability, alterations in the sodium/calcium channels, pro-inflammatory cytokines, and neurotransmitters (e.g., serotonin, dopamine, and glutamate) (88).

### Depression

Previous research has consistently found an increased prevalence of major depression disorder (MDD) and a higher risk of current depression in patients with migraines (34, 86, 141, 173, 174). For instance, a Korean hospital-based study reported that 36.3 % of migraine patients also had depression (175). Moreover, in an Italian multicenter study, 23.1 % of migraine patients also exhibited MDD (176). Furthermore, the results of a meta-analysis showed that, in comparison to non-migraineurs, migraine sufferers had a higher risk of being depressed (34). However, the odds of comorbid depression were reduced with increasing age, marriage, employment, and higher household income (34). Previous longitudinal research has found a bidirectional relationship between depression and migraine, with migraines predicting depression and depression predicting migraines (143, 174). The risk of current depression has also been found to be higher in migraine patients, compared to controls, and headaches appear to be independently associated with depression in the elderly (141). A number of studies have suggested that migraines can cause depression, with the severe pain from migraines lowering the patients' quality of life and leading to depressive symptoms (174, 177). Alternatively, migraine can be a manifestation or outcome of depression (178). Symptoms of pain are common in depressed people, and as a consequence some researchers have suggested that pain should be a component of depression (174, 179, 180), but the evidence to support this is not strong (174).

In a case-control study, the researchers not only found a general relationship between recurrent headaches and depression, but also a specific association between depression and migraine with aura (181). In another study, with a large sample, 28.5% of migraine sufferers were considered clinically depressed, while only 12.3% of those without migraines were clinically depressed (154). The mechanism behind the depression-migraine comorbidity, can be explained by heritability, genes [e.g., 5-hydroxytryptamine (5-HT) transporter genes and D2 receptor genes], neurotransmitters [e.g., serotonin, dopamine, and gamma-Aminobutyric acid (GABA)], the hypothalamic–pituitary–adrenal (HPA) axis, and the “neuro-limbic” pain network (88).

Although there are no studies which have failed to find a relationship between depression and migraine, there is a study that did not find an association between MDD and migraine (182). Nevertheless, that study had a number of methodological differences and limitations. Firstly, the patients studied were all relatively young and were not matched by sex. Secondly, the sample was not representative of the general population, or of a clinical setting, since the study only included young adults identified as being “high psychopathological risk,” using the general symptom scale (141). Finally, the recruitment of the clinical cases was based on the use of structured diagnostic interviews (SPIKE), which were originally designed for epidemiological studies and not specifically for diagnostic purposes (141).

### Attention Deficit Hyperactivity Disorder

There is very little research on the relationship between migraine and ADHD (85, 183). However, a cross-sectional study reported an increased prevalence of migraine in patients with persistent ADHD, compared with the general population (183). It has been shown that comorbid depression and anxiety were more prevalent in migraine patients with persistent ADHD, in comparison to those without migraines (85). Furthermore, a systematic review and meta-analysis found that there was a significant association between migraine and ADHD (184).

### Suicide

Migraine has also been shown to lead to an increased risk of suicidal behaviors, in both the clinical and general population, and among chronic migraineurs with and without aura (185, 186). The research with low bias ratings overwhelmingly supports a strong relationship between migraine and suicidal behaviors (34, 185). The role of depression in this relationship is unquestionable, with a clear bidirectional relationship between depression or anxiety disorders and migraines, which can increase the risk of suicide (34, 88).

There is also evidence to show migraines are associated with suicide attempts in migraine patients with aura, even after adjusting for major depression (141, 147). In population studies, migraine sufferers are between 2.2 and 4.0 times more likely to suffer from MDD, than non-migraineurs, and they are also at higher risk for suicide attempts, regardless of depression status (157). A meta-analysis showed a modest positive association between migraine and suicidal ideation (187). However, the complex interaction of factors underlying the relationships between migraine, suicide risk, and mood disorders deserves more scientific attention and research with strong methodological rigor (141).

## Limitations

The main limitation of this study that should be taken into consideration is that the current review is a narrative not a systematic review. Therefore, we cannot draw any precise conclusions due to the lack of the systematic search and meta-analysis. Furthermore, we only searched the PubMed database and restricted the search to only English language studies and those that were conducted on humans, so we may have missed some eligible articles. Finally, there were differing levels of evidence for the different sections covered in this review, with a number of the sections having extensive support in the literature and others less support (e.g., 5.1, 5.3, 5.7, 5.10, 5.11.2, 5.12.1, 5.12.2). Despite these limitations, our study highlights areas that need further work.

## Conclusions

The complex and multifactorial nature of migraine is reflected in the presence of a variety of risk factors and triggers agents. Furthermore, there is extensive evidence to indicate that various biological factors, especially hormones, genetic factors, and metabolic disorders, in addition to psychiatric and psychological factors are risk factors for migraine. Further work is needed to better understand the biological phenomena underlying migraine. The identification of the important biological, psychological factors and understanding the pathophysiological mechanisms can open new insights into the prevention, redesign of care pathways, management plans, and personalized therapy strategies.

## Author Contributions

MS, A-AK, and SS conceptualized and designed the study. PA, SK, SN, RM, HP, and MA-K drafted the initial manuscript. All authors reviewed the drafted manuscript for critical content and approved the final version of the manuscript.

## Funding

The present study was funded by Social Determinants of Health Research Center of Shahid Beheshti University of Medical Sciences, Tehran, Iran (Grant No. 24459).

## Conflict of Interest

The authors declare that the research was conducted in the absence of any commercial or financial relationships that could be construed as a potential conflict of interest.

## Publisher's Note

All claims expressed in this article are solely those of the authors and do not necessarily represent those of their affiliated organizations, or those of the publisher, the editors and the reviewers. Any product that may be evaluated in this article, or claim that may be made by its manufacturer, is not guaranteed or endorsed by the publisher.
